# As group size increases, individuals modify their vocal features to signal cooperation while remaining recognizable

**DOI:** 10.3389/fpsyg.2026.1765090

**Published:** 2026-06-24

**Authors:** Elisa Pellegrino, Volker Dellwo

**Affiliations:** Department of Computational Linguistics, University of Zurich, Zurich, Switzerland

**Keywords:** group size, recognizability, vocal accommodation, voice acoustics, voice individualization

## Abstract

**Introduction:**

Cooperation is essential to humans and manifests in speech through acoustic convergence, in which interlocutors’ voices become more similar. Yet convergence can be limited when signaling individuality is more important than aligning with others. In such contexts, speakers may adopt non-accommodative strategies to preserve their vocal identity and recognizability. Drawing on animal communication research—which shows that species in larger social groups exhibit greater vocal individuality—we test how group size (3 and 5 interactants) shapes the balance between remaining identifiable while still cooperating to support communication.

**Methods:**

In an interactive online game, players collaborated on a shared task while trying to recognize one another by voice, with the player recognized best receiving a reward. To assess how group size influences accommodation under these dual demands, we analyzed the speech of three players who participated in both game sessions. Acoustic features relevant to voice identity and amenable to accommodation—harmonicity, jitter, F0, formant dispersion, and duration—were extracted and reduced through Principal Component Analysis to two dimensions accounting for 52.9% of the variance. Following the identification of group size effects on these components, we assessed whether inter-speaker acoustic differences increased, decreased, or remained stable across conditions. Additionally, within-speaker variability was examined as a function of group size to determine whether observed changes in the five-player condition were driven by all speakers or only a subset.

**Results:**

In larger groups, accommodation was selective: players modulated their acoustic features, maintaining some while converging on others. No significant differences as a function of group size were observed for PC1, interpreted as reflecting maintenance and primarily associated with voice quality measures (harmonicity and jitter). In contrast, significant differences emerged for PC2, largely driven by F0 standard deviation and duration. These changes indicated reduced inter-speaker differences in the larger group, consistent with convergence. In the larger group setting, convergence was observed when two speakers showed mutual alignment and also shifted toward the speech patterns of a third participant, who remained comparatively stable in their acoustic behavior.

**Discussion:**

The pattern of selective and asymmetrical convergence indicates that speakers strategically balance the goals of cooperation and individuality, suggesting that recognizability demands also shape accommodation.

## 1 Introduction

Cooperation is a central aspect of human social interaction, and speech, together with dance and music, is one of the key media for signaling it (Dunbar, 2012; Leimgruber, 2022; Patel, 2018; Pearce et al., 2015; Podlipniak, 2022a, 2022b; Savage et al., 2021; Tarr et al., 2014). During social encounters, indeed, interlocutors adjust their speech behavior in response to others to manage social distance, control personal/social identity and regulate comprehension (Gasiorek, 2016b). These adjustments can take the form of convergence, divergence, or maintenance, and collectively refer to as accommodation (Giles, 2016). Convergence is considered the default communication move that interlocutors use to signal cooperation in interaction and support the communication (Leimgruber, 2022; Pardo et al., 2022). Speakers tend to rely on non-accommodative strategies (divergence and maintenance) when they wish to distinguish themselves, reinforce their own personal and/or social identity, make the communication difficult or counterbalance their interlocutor’s extreme speech patterns (Dragojevic et al., 2016). Because of this counter-cooperative nature of divergence and maintenance, they are sometimes referred to as the “dark side” of accommodative adjustments and are associated with having negative outcomes on the relation between interlocutors (Gasiorek, 2016a). In Pellegrino and Dellwo (2023) and in this study, we adopted a distinct perspective on divergence and maintenance. We proposed that non-accommodative strategies might be employed in cooperative contexts to preserve speaker-specific vocal characteristics and enhance individual recognizability. One such context is, for example, communication within larger groups. Research on animal communication has shown that vocal individuality tends to increase in species living in larger groups (Pollard and Blumstein, 2011) and within the same species as social network size expands (Mathevon, 2022). Psychological studies further suggest that individual and group payoffs resulting from each member’s behavior may influence cooperation in interaction (Capraro and Barcelo, 2015; Wu et al., 2020) and, plausibly, the tendency to adopt convergent communication strategies in larger groups. Building on these insights, in the current study and in Pellegrino and Dellwo (2023), we examined how group size affects speakers’ tendencies toward sounding cooperative through convergence for effective communication and enhancing their vocal individuality through divergence or maintenance for being recognized. To this purpose, we elicited naturally occurring forms of accommodation in players engaged in a cooperative game (Dominoes) within groups of varied sizes (three and five speakers), associated with a voice recognition task. The cooperative nature of the Dominoes game encouraged players to converge to communicate effectively and collaborate with each other to finish the game. At the same time, the recognition task required them to make their vocal identity distinctive, possibly through divergence and maintenance, as correctly identifying teammates by voice—and being recognized in return—was essential for winning the match (see par. 2.1 The game design). As outlined in Pellegrino and Dellwo (2023), the size of the two groups was guided by prior research indicating that listeners can reliably learn and recognize between four and six voices with minimal training (Legge et al., 1984; Yonan and Sommers, 2000; Kilgore and Chignell, 2006), a range also commonly used in established voice recognition and memory tests (4 voices in the Glasgow Voice Memory Test by Aglieri et al., 2017 and in the Greenwich Voice Recognition Test by Jenkins et al., 2021; 6 voices in the Jena Voice Recognition by Humble et al., 2022). Based on this evidence, we recruited one group of three and one group of five participants, assuming that recognizing two to four speakers would remain manageable, even under conditions with limited familiarization, no visual cues, and a non-typical separation between familiarization, training and testing phases.

How can we assess whether players tend to converge or enhance their vocal individuality when interacting in larger groups? There are multiple methods and features to evaluate these tendencies. For instance, in Pellegrino and Dellwo (2023) we first obtained i-vector speaker representations based on Mel Frequency Cepstral Coefficients (MFCCs) for the recording of each speaker in in- and out-of game sessions. Second, we measured vocal similarities between i-vector speaker representations obtained through probabilistic linear discriminant analysis (PLDA) from recordings of players in different group sizes (groups 3 and 5). Third, we tested the effect of individualization or cooperation on the recognition performance of an i-vector/PLDA recognition system in terms of Equal Error Rate (EER) when recordings of the individual players outside the game sessions were compared with those obtained in the larger and the smaller groups. The results indicated that speakers were more similar to one another in the larger groups—reflecting stronger cooperative vocal behavior—while overall recognizability of the same individuals declined when comparing recordings from the smaller and larger groups. However, the i-vector approach only showed that speakers become acoustically more similar during the interaction in the larger group but – because of their abstract nature – they do not reveal on which interpretable acoustic dimension speakers apply the changes. Further, this previous approach did not clarify whether the observed convergence resulted from mutual adjustments between speakers or from unidirectional adaptations of one player toward another.

To better understand how players balanced between vocal cooperation and individualization in the larger group, in the present study, we exchanged the i-vectors with interpretable acoustic dimensions. Practically, we selected parameters related to voice source characteristics, vocal tract resonances and dynamics articulation of speech, including fundamental frequency (F0) mean and standard deviation, voice quality (jitter and harmonicity mean and standard deviation), formant dispersion and word duration. These features capture voice individuality, are relevant for voice identity processing in humans (Kuwabara and Sagisaka, 1995; Perrachione et al., 2019; Kreiman et al., 1992; Baumann and Belin, 2010) and amenable to volitional modulations by individual speakers due to accommodation or other communicative demands (Levitan et al., 2012; Rahimi et al., 2017; Schweitzer et al., 2019). Considering that these features may be correlated and act together in the production and perception of speakers’ individuality, we analyzed the combined effect of these measures by means of a Principal Component Analysis (PCA). Through the PCA, we obtained the dimensions that contribute to the largest variance in the data and the features that are most representative for each dimension (see par.2.5.2). We expect that source-related features, such as F0 mean and voice quality features group together in one dimension, while resonances-related feature such as formant dispersion, and dynamic features such as duration and F0 standard deviation in two separate dimensions. Following the identification of group size effects on relevant PCA components, we assessed whether inter-speaker acoustic differences increased, decreased, or remained stable across conditions. Additionally, within-speaker variability was examined as a function of group size to determine whether observed changes in the five-player condition were driven by all speakers or only a subset. The results of the present study and those from Pellegrino and Dellwo (2023), taken together, will shed light on how speakers acoustically position themselves in interaction along the continuum between vocal cooperation and vocal individualization, using different sets of acoustic features and methods. This in turn gain a more comprehensive understanding of accommodative and individualized features in voice.

### 1.1 Hypotheses

Building upon Pellegrino and Dellwo (2023), we formulated two hypotheses concerning the effect of group size on vocal cooperation and individualization.

- *Cooperative Hypothesis*: If participants prioritize cooperation over competition in favor of being recognized, we hypothesize that the pattern of increased acoustic similarity in speakers’ i-vectors within group 5 (Pellegrino and Dellwo, 2023) would also emerge when examining the PCA dimensions derived from more interpretable acoustic features. In other words, we expect a reduction in the acoustic difference at the pair level from group *N* = 5 to *N* = 3, if the cooperative hypothesis holds.
- *Individualization Hypothesis*: If players’ incentive to maximize their recognizability in larger groups outweighs the drive to sound cooperative, we expect that the acoustic difference at the pair level will either increase from group *N* = 3 to *N* = 5 (indicating divergence) or remain comparable between the two groups (indicating maintenance).

Given that convergence, divergence, and maintenance are not mutually exclusive strategies (Gallois et al., 2005), and that different features can exhibit distinct patterns of accommodation within the same communicative context (Ostrand and Chodroff, 2021; Priva and Sanker, 2018; Sanker, 2016), individuals may simultaneously employ opposing acoustic strategies—sounding cooperative through converging on certain features while preserving their auditory recognizability through divergence or maintenance on others. In this study, we thus formulated a third hypothesis.

- *Selective Cooperative Hypothesis*: Players may selectively converge in certain acoustic dimensions while diverging or maintaining their individuality in others to strike a balance between cooperation and recognizability. If this hypothesis holds, we expect to observe a combination of patterns associated with both the cooperation and individualization hypotheses as the group size increases. Among the features examined in this study, it is plausible that individualization would be observed in more physiologically dependent voice source and resonance features, such as mean F0, jitter, harmonicity, and formant dispersion (Abercrombie, 1967). In contrast, cooperation is expected to manifest in more dynamic and malleable features, such as duration and F0 standard deviation.

No specific hypotheses can be stated about the driver of accommodation pattern at the pair level based on Pellegrino and Dellwo (2023), as the PLDA similarity score are not informative about who acted as the drivers of convergence.

## 2 Method

### 2.1 The game design

To create a controlled and meaningful experimental setup to investigate how group size influences the balance between sounding cooperative and recognizable, the design of the study was informed by linguistic and extralinguistic factors that have been shown to have an impact on vocal accommodation and voice recognition. A thorough description of these factors and how they were considered in the game design is available in Pellegrino and Dellwo (2023). A brief overview is given below:

- All participants in the study were females (see par. 2.2 Participants) and acted as both information giver and receiver. This choice has been made based on previous research indicating that gender and conversational role interact in a complex fashion in determining the degree and direction of vocal accommodation (Pardo et al., 2017).
- Participants played Dominoes in an interactive online playroom, accessible with video conferencing tools (see par. 2.3 Procedure). This provided a conducive environment for synchronized movements and real-time interactions. The ease of the Dominoes game mechanics, and the engaging online game environment were expected to promote vocal convergence among participants based on the positive impact of low cognitively demanding and high-engagement task on accommodation (Abel and Babel, 2017; Biro et al., 2022).
- The linguistic content of the interactional exchanges was designed to vary only in the number and colors of dots on the dominoes tiles (see par. 2.4 Speech Material). This controlled linguistic variation allows the researchers to control for the confounding effect of lexical properties and previous exposure on accommodation (Goldinger, 1998; Goldinger and Azuma, 2004).
- The participants played in two different group sizes, three and five players. The choice of these group sizes was based on voice recognition studies and voice memory tests suggesting that 4–6 voices are ideal to be learned with ease and short training (Kilgore and Chignell, 2006; Legge et al., 1984; Yonan and Sommers, 2000).
- The reward system was designed such that it encouraged players to focus on their vocal distinctiveness and clarity, as rewards were provided to the player who could be best recognized by their fellow players. It also supported active listening among the players as a separate reward was offered to the players with the highest recognition rates.

### 2.2 Participants

Five female Bernese German speakers ranging in age between 22 and 26 y.o. (mean 24.5) auditorily pre-screened for comparable pronunciation patterns were recruited as players. Participants were unacquainted before the experiment. One male Bernese German speaker (22 y.o.) acted as the experimenter. Participants declared they had no vision, no hearing, or linguistic impairment. Participants gave their informed consent to participate in the study and received monetary compensation for their participation. The study was conducted within the guidelines of the Ethics Committee of the Zürich University Faculty of Arts and Social Sciences.

### 2.3 Procedure

The step-by-step description of the procedure is based on Pellegrino and Dellwo (2023, p. 5, 6):

#### 2.3.1 Training sessions

Prior to the game sessions, all players were familiarized with the dominoes online playroom and the game mechanics with the support of the experimenter via ZOOM.

#### 2.3.2 Between the training and game sessions

After the training sessions, each participant was provided with the following information:

- Zoom invitation link that granted them access to the game session in groups of three and/or five players.
- Unique identification name represented by a letter from A to E. This unique identification name helped distinguish players across the two game sessions.
- A player number ranging from 1 to 5. The player number varied between the two game sessions for players who participated in both sessions. This variation was introduced to avoid any undue advantage for the individuals from group 3 in matching labels to voices in the session with 5 players.
- Cardholder number ranging from 1 to 5. Like the player number, the cardholder number varied between the two game sessions for participants who took part in both sessions.

#### 2.3.3 In-game sessions

On the experiment day, the players and the experimenter gathered on Zoom with their video cameras turned off. The experimenter then went over the game rules, shared the Domino playroom link in the chat, and prompted the players to unmute their microphones and join the playroom. The initial game session comprised three players, and the subsequent session included five players, with three from the initial session and two new players.

#### 2.3.4 Start game

The game begins with all players moving the first card from the cardholder to the hand area. Cards in the hand area remain hidden from other players (Step 1).

The player whose first card matches the number of dots on the card in the boneyard initiates the game (Step 2).

#### 2.3.5 First round of the game (familiarization – speaker identity revealed – no recognition test)

The player holding the turn greets the others (“Hello” – step 3), reveals her identity (e.g., “I am player one”- step 4), states the content of the card she has and the one she is searching for (“I have the dominoes stone with six yellow tiles, I am looking for the dominoes stone with three red dots – step 5). This process continues until all players have disclosed their identities.

#### 2.3.6 Following rounds of the game (testing – players’ identity omitted – recognition test)

The player who possesses a card matching the number of dots as the card in the boneyard begins the new round (Step 2).

The starting player greets the other participants (Step 3), states the content of the card she has and the one she is seeking (Step 5) but omits the identity disclosure step (Step 4 omitted).

Following that, the experimenter launches the Zoom poll (Step 6). All players participate in the poll by guessing the identity of the speaker holding the turn. The player who is currently holding the turn clicks on the option “I am the player” in the poll (Step 7). After all players voted, the experimenter shares the results of the poll (Step 8). However, no correct answer is provided. Instead, the poll results display the frequency with which each option was voted for by the participants.

In the subsequent rounds of the game, a new player takes her turn, and the procedure repeats until all players have played their cards.

At the end of the in-game session in group 5, participants are invited to fill out a socio-linguistic questionnaire about their language background, language/speech impairment, hearing level, musical/singing/acting skills.

#### 2.3.7 Post-game sessions

After the initial game sessions were completed, 1 month later, the five players who participated in the game were invited once again for post-game sessions. During these post-game sessions, the three players involved in both game sessions were asked to record the utterances they had produced during the matches with both three and five players. The remaining two players recorded only the utterances produced in the larger group. The post-game recordings were collected with the purpose of examining the speaker verification performance of the i-vector/PLDA system VOCALIZE (Pellegrino and Dellwo, 2023) and for future studies on voice recognition by human listeners when post-game recordings were compared to in-game recordings obtained in groups 3 and 5.

Throughout both the in-game and post-game sessions, the experimenter did not actively participate in playing the game. His responsibilities included exclusively recalling the game instructions and indicating the transition between different game rounds. For the audio recording of the speech performance during both the in-game and post-game sessions, the Zoom teleconferencing platform was used. Participants utilized their built-in microphones and headphones during the gameplay. To ensure that all participants’ audio streams were captured separately, the “Record a separate audio file for each participant” option was enabled in the Zoom recording settings. After the sessions were conducted, the experimenter saved the audio tracks locally on his local computer. These audio tracks were initially in .m4a format and were later converted to .wav format using Adobe Audition 2021. The conversion process ensured a standardized format of 32.000 samples/s and 16-bit quantization for all participants’ audio files.

### 2.4 Speech materials

The total speech corpus comprises 80 recordings of a 27–29-word long carrier texts in Bernese German, transcribed according to Dieth’s spelling (Dieth, 1986). An example of the game’s script in Bernese German transcribed according to Dieth’s spelling and its relative translation in English is provided in (1):

(1). Hallo. I bi d Spilerin 3. I ha dr Dominostei mit dä 3 rotä Pünkt. I sueche nachem Dominostei mit dä 2 grüene Pünkt. Het öpper dä Dominostei? Merci.

(eng. Hello. I am Player three. I have the dominoes card with three red tiles, and I am looking for the dominoes card with two green tiles. Who has this dominoes card? Thank you).

Of the 80 recordings, 15 were derived from the in-game session in group 3 (3 speakers * 5 game rounds), 25 in group 5 (5 speakers * 5 game rounds) and 40 from post-game sessions (15 from group 3 and 25 from group 5). Object of analysis for the present study are the utterances produced by the three participants playing in both the in-game sessions (group 3 and 5). We will henceforth refer to three speakers to as SpeakA, SpeakB and SpeakC.

### 2.5 Data analysis

#### 2.5.1 Acoustic analysis of the corpus

The speech corpus produced by the three speakers in the in-game sessions in group 3 and 5 were segmented and labeled using Munich Automatic Segmentation Systems (MAUS – Kisler et al., 2017; Schiel, 1999). To guarantee maximum precision, the automatic segmentation and labeling of signal in Bernese Swiss German were manually checked and corrected using Praat (version 6.1.52; Boersma et al., 2026). The corrections concerned the labeling of a-vowels in au-diphthongs (e.g., [ae] instead of [a]), velar nasals (e.g., [n] instead of [N]) and near-close near-front rounded vowel (e.g., [u] in place of [ʏ]). Every phonologically annotated word of the corpus was automatically extracted by means of a Praat script, for a total of 749-word signals (Table 1 – Before outlier detection).

**Table 1:** Number of word utterances by the three speakers in in-game session in group 3 and 5, before and after outlier detection for acoustic analysis.

	Before outlier detection		After outlier detection	
	Group 3	Group 5	Group 3	Group 5
Speak A	118	122	111	116
Speak B	127	134	122	129
Speak C	126	122	124	114
Total	371	378	357	359

The analysis is conducted at the word-level to enable future studies on how acoustic accommodation influences perceived convergence and to ensure comparability with previous research (see a.o. Babel et al., 2013; Cohn et al., 2023; Pardo, 2013) through the consistent choice of units.

To avoid measurement artifacts particularly occurring in non-harmonic sections of speech, an algorithm based on harmonicity detection was applied to reduce the signal to harmonic sections only. For every word-signals deprived by the non-harmonic parts, we used the standard settings of the Praat software to extract a set of acoustic voice features with documented behavioral relevance for person identity processing (Kuwabara and Sagisaka, 1995; Perrachione et al., 2019; Kreiman et al., 1992; Baumann and Belin, 2010) and being prone to inter-speaker mutual adjustments (cf. Pardo et al., 2022 and related special issue papers for a review of recent findings): mean and standard variation of fundamental frequency, the mean and standard variation of harmonicity, jitter, duration, long term F1, F2, F3 and F4 to obtain formant dispersion. Word entries in the data frame presenting outliers detected through multivariate Robust Mahalanobis distances were excluded from the analysis. As a result of outlier removal, 5% of observations were removed, and the acoustic analyses were carried out on 716 out of 749-word signals (Table 1 –After outlier detection).

#### 2.5.2 Principal component analysis

The selected acoustic features related to voice source characteristics (F0 mean, jitter), vocal tract resonances (formant dispersion) and dynamics articulation of speech (word duration, F0 standard deviation) (1) act together to influence voice individuality (Kuwabara and Sagisaka, 1995; Perrachione et al., 2019; Kreiman et al., 1992; Baumann and Belin, 2010), (2) trigger accommodative adjustments (cf. Pardo et al., 2022 and related special issue papers for a review of recent findings) and (3) are significantly correlated (Bartlett test; *x*^2^ = 1106.087; *p* < 0.001). For these reasons, we computed a full principal component analyses (PCA) including mean and variability measures for F0 and harmonicity, jitter, word duration and formant dispersion. Following the Kaiser criterion (Kaiser, 1960), we retained PCs that have eigenvalues greater than 1. Following usual practice, acoustic variables with contribution larger than the expected average contribution were considered as important in contributing to the component (Abdi and Williams, 2010). Analyses resulted in two principal components (PC1 and PC2) with eigenvalues greater than 1 (for details see par. 3.1. Results PCA).

#### 2.5.3 Operationalization of accommodation

To examine the dynamics of vocal cooperation, individualization or selective accommodation as function of group size, we tested the effect of group size on principal components with eigenvalues greater than 1 using Linear Mixed-Effects Models implemented in the *lme4* R package. The random-effects structure initially included both item and game round as random intercepts; however, this specification resulted in a singular fit. Consequently, we retained only item as a random intercept in the final models. A non-significant effect of group size on the relevant components is interpreted as evidence of maintenance, i.e., no systematic acoustic modification in players’ behavior as a function of group size. When a significant effect of group size was observed, we computed estimated marginal means (EMMs) for each combination of group size (Group 3 vs. Group 5) and speaker identity (SpeakA, SpeakB, SpeakC), followed by pairwise comparisons with Tukey-adjusted *p*-values. Based on these analyses, we derive a measure of accommodation at the pair level (henceforth accommodation within a pair) by comparing the estimated contrasts between each pair of speakers in Group 3 and Group 5. Lower estimates in Group 5 relative to Group 3 (baseline) were interpreted as evidence of convergence, whereas larger differences in Group 5 indicated divergence; no differences were taken as evidence of maintenance. Moreover, we examined within-speaker variation across Group 3 and Group 5 us to identify which player(s) were more acoustically malleable and therefore more likely to be the driver of the observed modifications at the pair levels when moving from Group 3 to Group 5.

## 3 Results

### 3.1 Principal component analysis

**Figure 1:**
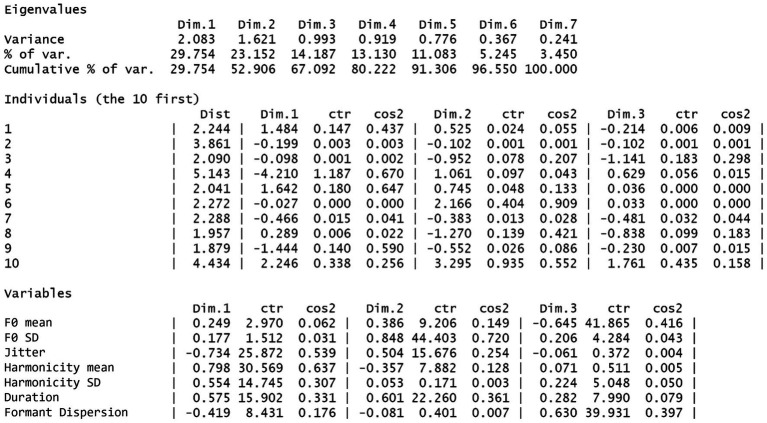
Results of the principal component analysis (PCA). The top panel reports eigenvalues, percentage of explained variance, and cumulative variance for each dimension. The middle panel presents the coordinates, contributions (ctr), and squared cosines (cos^2^) of the first 10 individuals on the first three principal components. The bottom panel shows the loadings (coordinates), contributions, and cos^2^ values of the acoustic variables across the same dimensions, indicating their relative importance in defining each component.

Analyses resulted in two principal components (PCs) with eigenvalues greater than 1. These components accounted for 52.9% of the cumulative acoustic variance in the data (Figure 1).

**Figure 2:**
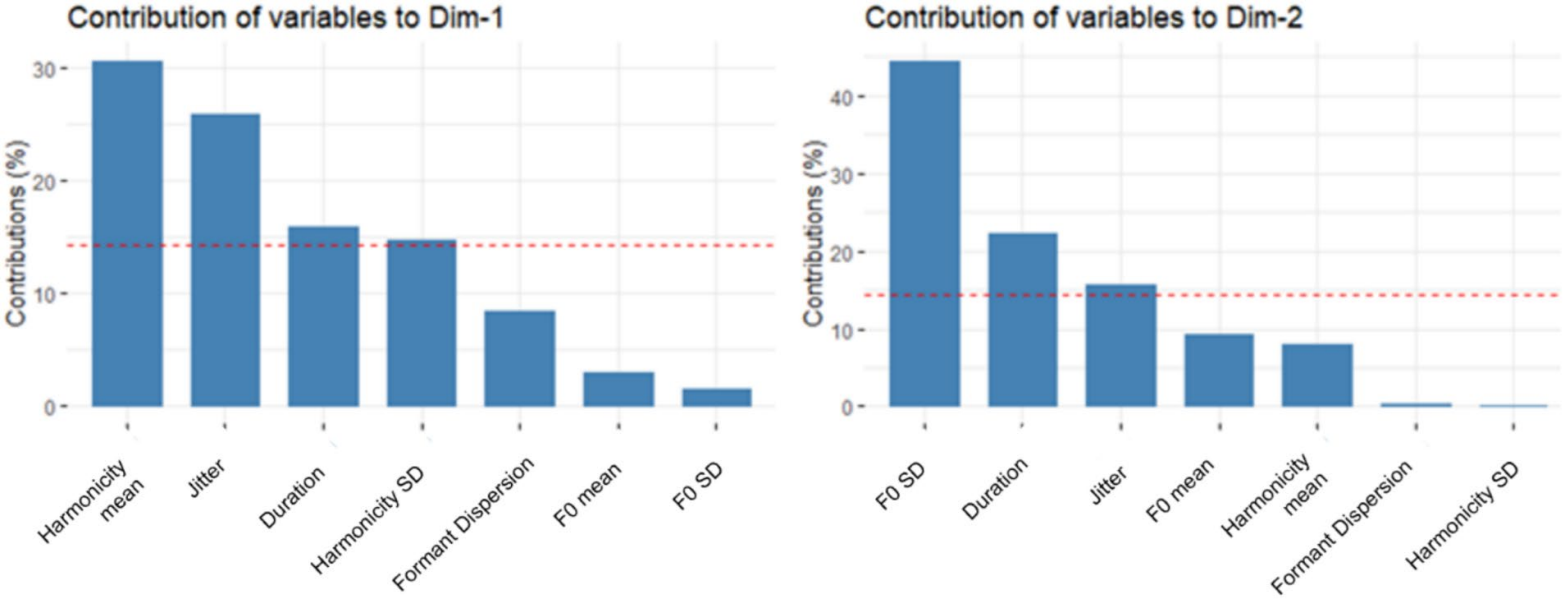
Total contribution (in percentage) of individual variables to the first dimension (left) and the second dimension (right). The red dashed line on the graph above indicates the expected average contribution (14.3%) given the seven variables.

As shown in Figure 2 (left and right), the distribution of variables in relation to weight across the first two components indicates, in partial accordance with the predictions, that voice source features and the more dynamic and malleable features group in two different dimensions. Harmonicity mean and jitter (but not F0 mean), indeed, predominated the first dimension, while F0 standard deviation and word duration played a more prominent role in the second component. Formant dispersion may contribute to dimensions with eigenvalues lower other 1; therefore, they are disregarded for the purpose of the present analysis.

### 3.2 Results on accommodation

**Figure 3:**
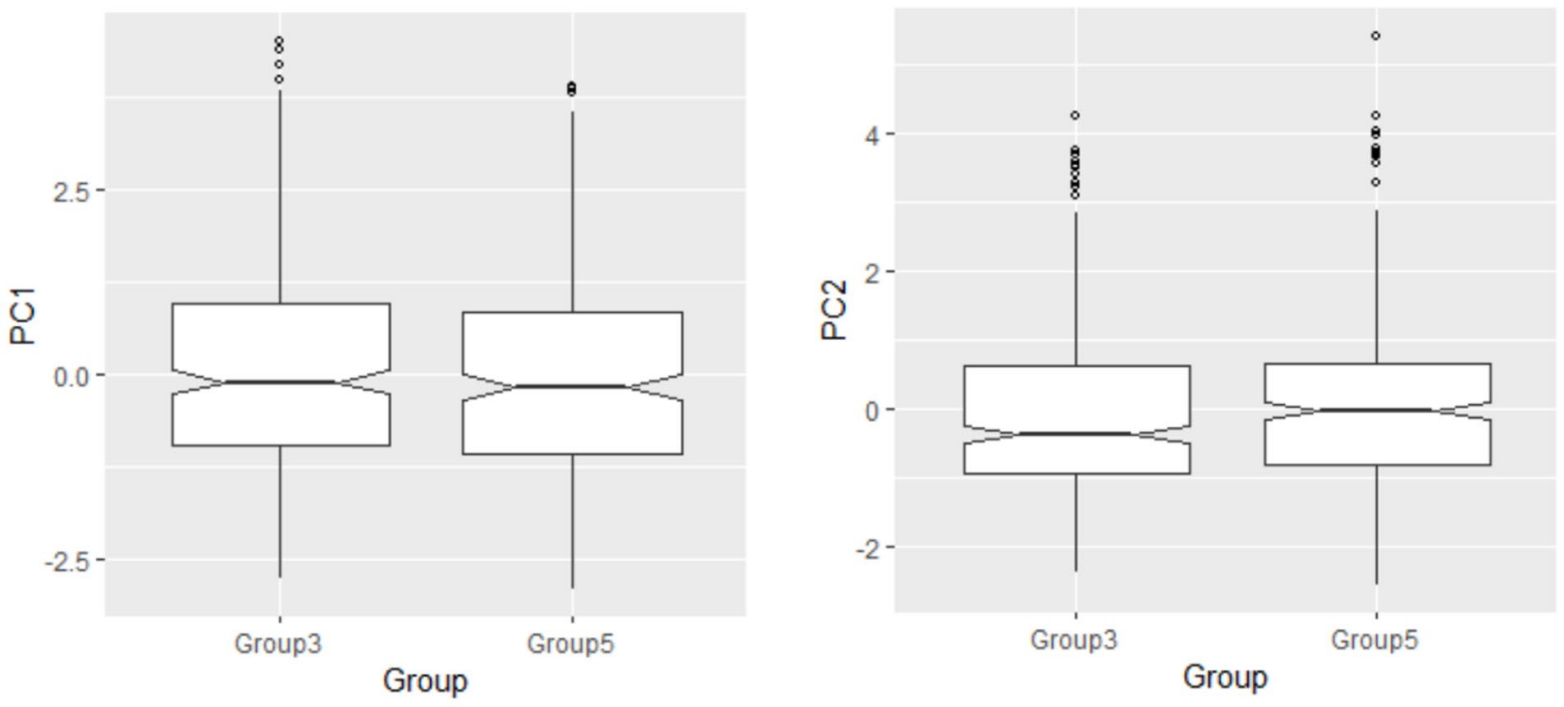
Distribution of PC1 (left) and PC2 (right) by group size.

Figure 3 (left panel) shows that the distribution of PC1 values between the two groups appears to be comparable, while for PC2 (Figure 3, right panel), on average, Group 5 demonstrates higher values than Group 3. The results of statistical analysis showed that the effect of group size on PC1 was not significant (*x*^2^ = 1.8348, *p* = 0.1842), while it was significant for PC2 (*x*^2^ = 12.348, *p* = 0.030).

**Figure 4:**
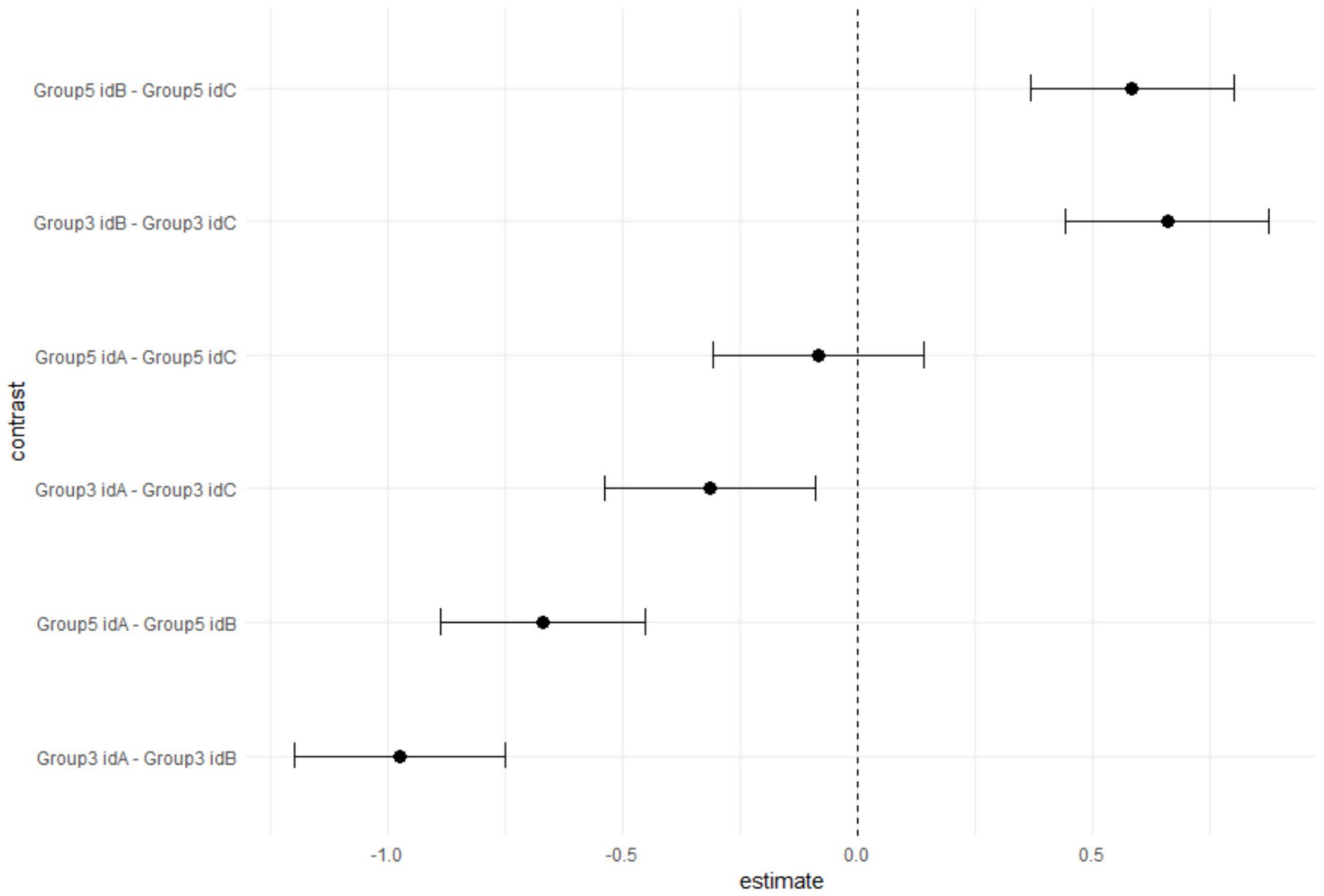
Point plots showing the EMM contrast by pair speakers (SpeakA-SpeakB, SpeakA-SpeakC) and group size. Error bars represent the standard error.

A comprehensive view of acoustic changes in PC2 at the pair level as function of group size is offered in Table 2 and Figure 4. The former presents the pairwise comparison estimates by speaker and group, while the latter plots the pairwise differences derived from these contrasts.

**Table 2:** Pairwise comparisons of Group × Speaker (Tukey-adjusted).

	Group3 SpeakA	Group5 SpeakA	Group3 SpeakB	Group5 SpeakB	Group3 SpeakC	Group5 SpeakC
Group3 SpeakA	−0.5451	0.0148	<0.0001	<0.0001	0.0691	0.0013
Group5 SpeakA	−0.3731	−0.1720	<0.0001	<0.0001	0.9953	0.9770
Group3 SpeakB	−0.9734	−0.6003	0.4283	0.9880	<0.0001	<0.0001
Group5 SpeakB	−1.0428	−0.6697	−0.0694	0.4977	<0.0001	<0.0001
Group3 SpeakC	−0.3144	0.0587	0.6589	0.7284	−0.2307	0.7895
Group5 SpeakC	−0.4578	−0.0846	0.5156	0.5851	−0.1433	−0.0873

Diagonal values represent estimated marginal means (emmeans). Lower triangle shows pairwise comparison estimates (earlier vs. later condition). Upper triangle reports Tukey-adjusted *p*-values.

**Figure 5:**
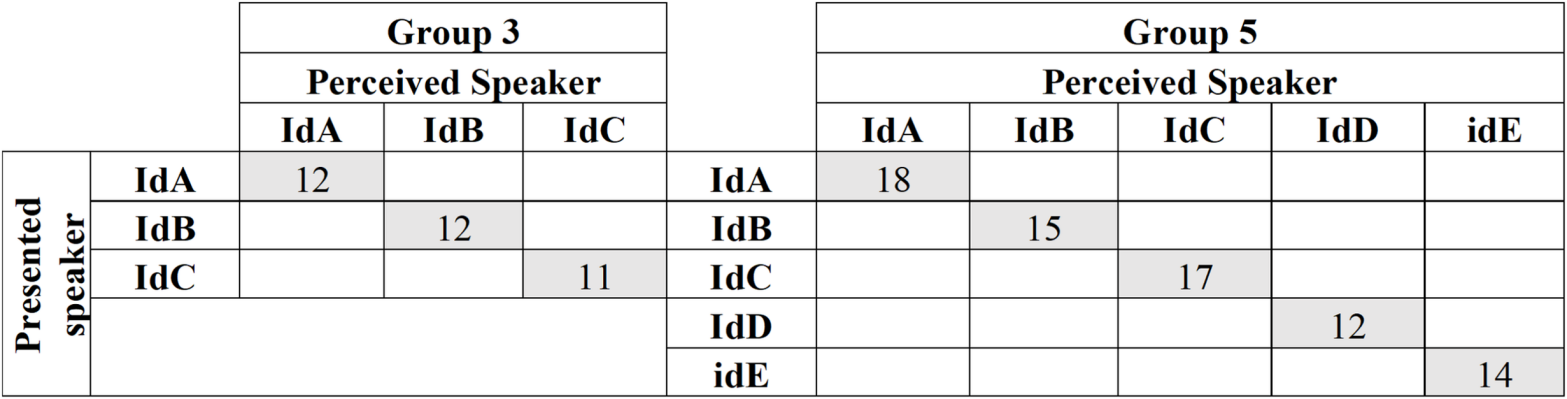
Confusion matrices showing presented and perceived speakers across game sessions played in group 3 and 5 (Pellegrino and Dellwo, 2023, p. 9). Numbers show correct identifications out of 12 attempts in group 3 and 20 in group 5. IdA stands for SpeakA, IdB stands for SpeakB, idC stands for SpeakC, idD stands for SpeakD and iDE stands for SpeakE.

As inferable from Table 2 and Figure 5, the acoustic differences decrease from Group 3 to Group 5 primarily for contrasts involving SpeakA. Specifically, Table 2 reports that the contrast between SpeakA and SpeakB decreases from −0.9734 in Group 3 to −0.6697 in Group 5, and the contrast between SpeakA and SpeakC decreases in magnitude from −0.3144 to −0.0846. Conversely, the difference between SpeakB and SpeakC remains relatively stable across group sizes (0.6589 in Group 3; 0.5851 in Group 5). Table 2 also permits to examine within-speaker variation as function of group size which is essential to understand which speaker(s) are more acoustically malleable and possibly drive the accommodation pattern observed at the pair level. Within this context, SpeakB exhibits the most stable and conservative behavior across group sizes (Group 3: −0.4283; Group 5: −0.4977; *p* = 0.9880). In contrast, SpeakA shows the largest and only statistically significant change increasing from −0.5451 to −0.1720 (*p* = 0.0148), indicating substantial acoustic adjustment in the larger group. SpeakC also shows an increase in PC2 (from −0.2307 to −0.0873), suggesting a shift in the same direction, although this change does not reach statistical significance (*p* = 0.7895). Taken together, these results suggest that the observed changes in Group 5 in PC2 is primarily driven by SpeakA, and – to a lesser extent – by SpeakC. SpeakB, instead, remains comparatively stable. The upward shifts in PC2 for SpeakA—and marginally for SpeakC—suggest an acoustic approximation toward SpeakB. Finally, the reduced acoustic difference between SpeakA and SpeakC in Group 5 may reflect either mutual convergence or an asymmetric process in which both speakers accommodate toward SpeakB; these interpretations are not mutually exclusive.

## 4 Discussion

In this study we investigated how individuals reconcile through voice the opposing needs of signaling cooperation for the sake of communication success and enhancing their individuality for the sake of recognizability. Methodologically, we expanded previous research that quantified and showed patterns of acoustic convergence in terms of abstract i-vector speaker representations (Pellegrino and Dellwo, 2023). In the present investigation, we examined the accommodative behavior of the three speakers based on a combination of interpretable acoustic features connected to vocal source and resonance characteristics (e.g., F0 mean, harmonicity mean and variation, jitter, formant dispersion) as well as more malleable, dynamic features (e.g., duration, F0 variation), reduced through Principal Component Analysis to two major dimensions explaining 52.9% of the variance in the data.

One key finding of this study is that the observed patterns of accommodation in a larger group size is selective. Maintenance is observed largely for PC1, predominated by voice quality features (jitter and harmonicity mean). Convergence, instead, was documented for PC2, predominated by F0 standard deviation and duration. Beyond confirming the granularity of accommodation (cf. Pardo et al., 2022), these results support the selective cooperative hypothesis (3rd hypothesis of our study): in cooperative situations requiring enhanced recognizability, speakers employ opposing accommodative strategies simultaneously, possibly depending on the acoustic characteristics of their interactants they support a strategy of disaffiliation (observed for voice quality features, i.e., harmonicity and jitter) and affiliation (observed for dynamic features, i.e., word duration and F0 standard deviation). A complementary view on selective accommodation is offered by some recent philosophical work contending, on a broader theoretical level, that individuality and cooperation may not constitute opposing ends of a continuum. Rather, individuality may be understood as emerging within collective systems, as a structured feature of social coordination (Griesemer and Shavit, 2023). In this sense, maintaining distinct voice quality features while converging on more flexible prosodic cues may reflect a division of labor, rather than a trade-off, across acoustic dimensions that supports both recognizability and coordination in interaction.

One possible explanation for the differential accommodative patterns between voice quality features on one hand, and duration and F0 standard deviation on the other hand, is that the former show maintenance because they are a “quasi-permanent” attribute of individual speakers (Abercrombie, 1967, p. 91). This characteristic can be partly explainable in terms of speaker’s physical characteristics and partly of their linguistic, social and cultural embedding (Kreiman et al., 2018). While this plausible explanation supports the view of voice quality as an index of speaker’s whole individuality, it is equally important to recognize that voice quality also serves as a rich phonetic resource through which speakers index group membership, express affect and take stances in interaction (Podesva and Callier, 2015). Individuals, indeed, volitionally modulate their voice quality to disguise their identity or mimicking others, albeit with detrimental effects on voice recognizability (Perrot et al., 2007). Furthermore, research has shown that voice quality features (including but not limited to jitter and harmonicity examined in this paper) are subject to both convergence and divergence, similar to other acoustic features, with consequences on the perception of interlocutors’ social behavior and dialog success (Levitan et al., 2012; Rahimi et al., 2017; Schweitzer et al., 2019). In addition, voice quality can be a marker of social group identity, for example, in the well-known phenomenon of ‘vocal fry’ according to which predominantly female speakers of central Northern American English varieties are characterized by an overtly strong use of voice qualities with considerable amounts of irregularity in periodicity. Such speakers are typically able to control the degree of irregularity depending on situation. It seems feasible that in social groups of speakers applying ‘vocal fry,’ a large degree of accommodation of this feature may occur. In view of this, interpreting maintenance on PC1 as a means for speakers to uphold their personal voice quality patterns for the sake of recognizability is a plausible and valid explanation in the context of this study. However, given the limited size of the dataset, more research—particularly with larger samples—will be necessary to examine the selective cooperative hypothesis (maintenance in voice quality and convergence on F0 variation and word duration) in greater depth and to more fully understand the underlying motivations and implications of speakers’ vocal behavior during communication.

Another notable finding of this study is the asymmetrical nature of accommodation in a larger group, where not all speakers reciprocated accommodative moves. Pair-level convergence was predominantly driven by SpeakA, who adjusted her behavior toward both SpeakB and SpeakC, while SpeakB exhibited little to no variation in acoustic behavior across game sessions. What might explain these individual differences in accommodation? Although the game was designed to minimize linguistic and extralinguistic variability among speakers, one key distinction between the three players lay in their musical training. According to the sociolinguistic questionnaire administered after the game sessions, SpeakA reported extensive musical experience, including 20 years of singing and several years of stage performance. By contrast, SpeakB and SpeakC had little to no specified musical training. Determining the exact role of SpeakA’s musical background in her greater propensity to converge is challenging. However, existing evidence suggests that musical training enhances speech perception and production abilities, including sensitivity to converging speech (McKay, 2021; Tsang et al., 2018). Furthermore, research indicates that vocal training, specifically singing, is a stronger predictor of speech imitation ability compared to instrumental training (Christiner and Reiterer, 2013). Considering this evidence, along with the absence of specific cognitive testing in this study, we can only speculate that SpeakA’s long-term singing experience provided her with enhanced auditory working memory and vocal flexibility. These abilities, together with likely more developed musical aptitude may have made her more attuned to pitch and rate features from other players, increasing her propensity to converge in these acoustic features (for research about musical aptitude and prosody discrimination, see Vigl et al., 2024). Conversely, SpeakB and SpeakC, lacking such training, may have been less sensitive to these features and less prone to convergence. In summary, this could suggest that higher musical skills not only interact with social skills (Burak and Baş, 2024; Voitova et al., 2025) but may also be associated with more effective accommodation skills. The literature on accommodation (e.g., Giles, 2016; Giles et al., 2023) has consistently indicated that greater accommodation tends to be linked with improved social integration. If these patterns were to extend to the present context, it might imply that individuals with higher musical aptitude could also demonstrate enhanced social integration, potentially mediated by more refined speech accommodation behavior. While this interpretation is theoretically grounded and consistent with existing literature, it nonetheless requires further empirical validation through more targeted and systematic investigation.

Another interpretation of the findings on between speaker variability in convergence considers the accommodative and non-accommodative strategies of the three speakers in relation to situational factors, particularly the degree of correct recognition in the larger group as obtained in Pellegrino and Dellwo (2023). In group 5, SpeakA and SpeakC, the most cooperative players, were correctly recognized 18 and 17 times out of 20, respectively, while Speak B, the most individualistic player, was correctly recognized only 15 times.

Evidence suggests that the strategic context, along with the individual and group payoffs linked to members’ behaviors, can influence cooperation in larger groups (Capraro and Barcelo, 2015; Wu et al., 2020). In this study, the higher rate of correct recognition for SpeakA and SpeakC may have encouraged cohesive and affiliative behaviors, leading to a measurable decrease in inter-speaker acoustic difference. Conversely, SpeakB’s lower rate of correct recognition might have prompted her to minimize acoustic variability to preserve her recognizability. It is difficult to draw conclusions from small identification numbers but assuming that these numbers replicate in larger studies, they would mean that recognizability is a key factor modulating the accommodative strategies interlocutors enact in conversation: the more recognizable a speaker is, the more likely they are to show affiliation and cooperation through convergence.

To sum up, the findings from the current study and those from Pellegrino and Dellwo (2023) collectively suggest that interactions within larger groups of unfamiliar speakers represent a specific communicative context where individuals simultaneously engage in signaling in-group cooperation while also seeking to maximize their own vocal distinctiveness. Both studies observed evidence of convergence, but – as revealed from the present study – this phenomenon is asymmetrical and selective. Some speakers, like SpeakA and SpeakC, adapted their behavior to achieve cooperation [reflected in convergence in PC2, MFCC (Pellegrino and Dellwo, 2023)] and effective recognition (reflected predominantly in maintenance in PC1). In contrast, SpeakB leaned toward prioritizing recognizability [evidenced by maintenance in both PC1, PC2 and possibly MFCC (Pellegrino and Dellwo, 2023)], as her recognizability was challenged.

The pattern of selective and asymmetrical convergence observed in this exploratory dataset suggests that individuals are sensitive to the competing demands of signaling cooperativeness while maintaining recognizability. Speakers appear to adjust their speech behavior in idiosyncratic ways, plausibly balancing or prioritizing between these conflicting goals. From an evolutionary perspective, this overall flexibility may reflect the coevolution of cooperation and individual differentiation, whereby pressures for coordination in social interaction coexist with pressures for maintaining distinct, recognizable identities (Hochberg et al., 2008). By examining the dynamic interplay between vocal accommodation and recognizability demands, this study provides a novel interpretative framework for understanding why interactants affiliate in some vocal features while disaffiliating in others. Given the limited sample size of the present research, and the dyadic interpretation of a multi-party setting, replication studies are essential to establish the generalizability of the findings and to deepen our understanding of how individuals in groups navigate social interactions—balancing cooperation with the maintenance of individual identity through vocal behavior.

## Data Availability

The raw data supporting the conclusions of this article will be made available by the authors, without undue reservation.
